# Social Predictors of Continued and Indoor Smoking Among Partners of Non-smoking Pregnant Women: The TMM BirThree Cohort Study

**DOI:** 10.2188/jea.JE20200313

**Published:** 2021-12-05

**Authors:** Keiko Murakami, Mami Ishikuro, Fumihiko Ueno, Aoi Noda, Tomomi Onuma, Taku Obara, Shinichi Kuriyama

**Affiliations:** 1 Tohoku Medical Megabank Organization, Tohoku University, Miyagi, Japan; 2 Graduate School of Medicine, Tohoku University, Miyagi, Japan; 3 Department of Pharmaceutical Sciences, Tohoku University Hospital, Miyagi, Japan; 4 Department of Disaster Public Health, International Research Institute of Disaster Science, Tohoku University, Miyagi, Japan

**Keywords:** continued smoking, indoor smoking, Japan, partners, pregnant women

## Abstract

**Background:**

Secondhand smoke (SHS) from partners is a major source of exposure for non-smoking women. However, epidemiological studies have rarely examined social factors associated with continued and indoor smoking among pregnant women’s partners.

**Methods:**

We analyzed data on 6,091 partners of non-smoking pregnant women in the Tohoku Medical Megabank Project Birth and Three-Generation Cohort Study. Partners’ age, education, income, workplace SHS exposure (*almost never or sometimes*, *almost every day*), and pregnant women’s smoking history (never, quit before pregnancy awareness, quit after pregnancy awareness) were used as social factors. Multiple logistic regression analyses were conducted to examine the associations of social factors with partners’ continued smoking and indoor smoking.

**Results:**

Among 2,432 smoking partners, 2,237 continued to smoke after pregnancy awareness. Workplace SHS exposure was associated with increased risk of partners’ continued smoking: the odds ratio of workplace SHS exposure *almost every day* compared with *almost never or sometimes* was 2.08 (95% confidence interval, 1.52–2.83). Women’s quitting smoking after—but not before—pregnancy awareness was associated with decreased risk of partners’ continued smoking: the odds ratio of women’s quitting after pregnancy awareness compared with never smoking was 0.57 (95% confidence interval, 0.40–0.80). About one-third of partners who continued to smoke did so indoors. Older age, lower education, workplace SHS exposure, and women’s quitting smoking after pregnancy awareness were associated with increased risk of partners’ indoor smoking.

**Conclusions:**

Workplace SHS exposure and pregnant women’s smoking history were associated with continued smoking and indoor smoking among partners of non-smoking pregnant women.

## INTRODUCTION

Growing evidence indicates that secondhand smoke (SHS) exposure during pregnancy has negative consequences on pregnancy and infant outcomes, such as reduced birth weight, stillbirth, and congenital anomalies.^1^^–^^3^ As SHS from partners is a major source of exposure for non-smoking women,^4^^,^^5^ partners’ smoking cessation is a priority in protecting pregnant women from SHS exposure. If these partners have difficulty with smoking cessation, refraining from smoking indoors is the second-best option.^4^^,^^5^ To design effective interventions for reducing SHS exposure during pregnancy, it is important to clarify factors associated with continued smoking and indoor smoking among the partners of non-smoking pregnant women.

Social factors, such as educational level, SHS exposure, and partners’ smoking status, may be differentially associated with continued smoking in different groups. Among pregnant women, having lower levels of education, higher exposure to SHS, and a smoking partner have been shown to be associated with increased risk of continued smoking,^6^ but these associations have not been observed among the general adult population.^7^ Qualitative research has identified barriers and facilitators to smoking cessation among the partners of pregnant/postpartum women: smoking being an integral part of everyday life, becoming and being a father, the couple’s relationship, perceptions of the risks of smoking, and their harm reduction and quitting strategies.^8^ This result suggests that social factors, such as educational level, SHS exposure, and pregnant women’s smoking status, are associated with continued smoking among their partners. However, epidemiological studies have rarely examined these associations among the partners of pregnant women.^9^^,^^10^

There is a lack of available data on the prevalence of indoor smoking and the factors potentially associated with indoor smoking among pregnant women’s partners who continue to smoke. Among fathers living in the same home with their children, attitudes and knowledge, cultural and social norms, gender power relations, and shifting perceptions and responsibilities related to fatherhood have been reported to influence views on the creation of a smoke-free home.^11^ However, there is little epidemiological evidence on the social factors associated with smoking partners’ indoor smoking before the baby is born.

Considering the above circumstances, we conducted an epidemiological study to examine the social factors associated with continued smoking and indoor smoking among the partners of non-smoking pregnant women in Japan, where SHS exposure among non-smoking pregnant women is a serious public health concern because of the large difference in smoking prevalence between men and women.^12^

## METHODS

### Study population

**Figure 1.**
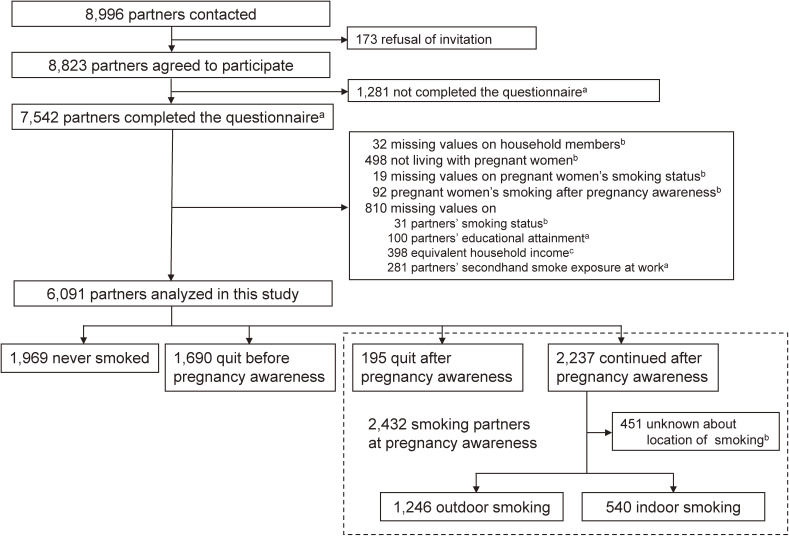
Flow diagram of participants in the present analysis of the Tohoku Medical Megabank Project Birth and Three-Generation Cohort Study. ^a^Data on partners’ educational attainment and secondhand smoke exposure at work were obtained using the questionnaire administered for partners at enrollment. ^b^Data on living with pregnant women, pregnant women’s smoking history, partners’ smoking status, number of household smokers, and number of smokers inside the residence were obtained using the questionnaire administered for pregnant women in early pregnancy. ^c^Data on equivalent household income were obtained using the questionnaire administered for pregnant women in middle pregnancy.

Data were obtained from the Tohoku Medical Megabank Project Birth and Three-Generation Cohort Study (TMM BirThree Cohort Study), which has been described elsewhere.^13^ Pregnant women and their family members, including the women’s partners (the fathers of the fetuses), were contacted in obstetric clinics or hospitals when they scheduled their deliveries from 2013 to 2017. Approximately 50 obstetric clinics and hospitals in Miyagi Prefecture participated in the recruiting process. Tohoku University Tohoku Medical Megabank Organization established seven community support centers in Miyagi Prefecture as local facilities for the voluntary recruitment and health assessment of the participants.^14^ Trained genome medical research coordinators were stationed in each clinic, hospital, and community support center to provide information on the TMM BirThree Cohort Study to potential participants and to receive a signed informed consent form from each participant. Of the 8,996 partners of pregnant women who were contacted, 8,823 agreed to participate in the study, and 7,542 completed the questionnaire (response rate: 85.5%). Of these respondents, 641 were excluded: 32 with missing values on household members, 498 who were not living with pregnant women, 19 with missing values on pregnant women’s smoking status, and 92 pregnant women’s smoking after becoming aware of their pregnancy. After further excluding 810 partners with missing values on smoking status, education, income, or workplace SHS exposure, the remaining 6,091 partners were included in the present study. Figure 1 shows the flow diagram of the study. The TMM BirThree Cohort Study protocol was reviewed and approved by the Ethics Committee of Tohoku University Tohoku Medical Megabank Organization (2013-1-103-1).

### Measures

Data on all variables used in the analysis were obtained using questionnaires, which are described in detail in eTable 1. Partners’ age was categorized into four groups: ≤29, 30–34, 35–39, and ≥40 years. Partners reported their educational attainment, which was divided into three categories: high school or lower (elementary, junior high school, or senior high school), college (2-year college or special training school), and university or higher (university or graduate school). Partners reported their workplace SHS exposure as how often they were exposed to someone else’s cigarette smoke at work in the past year. The responses were dichotomized as *almost never or sometimes* and *almost every day*. Pregnant women reported their smoking status, which was divided into three categories: never smoked, quit before becoming aware of their pregnancy, and quit after becoming aware of their pregnancy. Pregnant women selected total annual household income from seven categories: ≤1.99, 2.00–3.99, 4.00–5.99, 6.00–7.99, 8.00–9.99, 10.00–11.99, and >12.00 million Japanese yen (JPY). Equivalent household income was calculated as household income divided by the square root of the number of household members,^15^ and the resulting value was categorized into four groups: ≤1.99, 2.00–2.99, 3.00–3.99, and >4.00 million JPY, which corresponded approximately to quartiles.

Pregnant women reported their partners’ smoking status, the number of household smokers, and the number of people who smoked inside their residence. Partners’ smoking status was categorized as never smoked, quit before becoming aware of the pregnancy, quit after becoming aware of the pregnancy, and continued after becoming aware of the pregnancy. Partners’ indoor smoking was defined as present when partners continued smoking after becoming aware of the pregnancy and pregnant women reported that there were any smokers inside their residence. If partners continued smoking after becoming aware of the pregnancy and there were no smokers inside the pregnant women’s residence, their partners were coded as smoking outdoors. Because it was impossible to identify who smoked in the residence when there were ≥2 household smokers, we decided to analyze social factors associated with indoor smoking only among partners who continued to smoke and lived with no other household smokers (ie, when there was only one household smoker).

### Statistical analysis

Multiple logistic regression analyses were conducted to examine the associations of partners’ age, educational attainment, equivalent household income, workplace SHS exposure, and the pregnant women’s smoking history with continued smoking among the partners who had been smokers when becoming aware of the pregnancy. Odds ratios (ORs) and 95% confidence intervals (CIs) were calculated for each factor adjusting for partners’ age, as well as all other factors. Among partners who continued to smoke after becoming aware of the pregnancy, Multiple logistic regression analyses were conducted to examine the associations of partners’ age, educational attainment, equivalent household income, workplace SHS exposure, and pregnant women’s smoking history with indoor smoking.

All analyses were conducted with SAS version 9.4 (SAS Institute Inc., Cary, NC, USA). For all analyses, a two-tailed *P* value <0.05 was considered statistically significant.

## RESULTS

Table 1 shows the characteristics of the partners of non-smoking pregnant women. Among these partners, 24.5% were in their 20s, 40.9% had a university education or higher, 48.0% were exposed to SHS at work, and 11.5% were married to women who had quit smoking after becoming aware of their pregnancy. As for partners’ own smoking status, 32.3% had never smoked, 27.7% had quit smoking before becoming aware of the pregnancy, 3.2% had quit after becoming aware of the pregnancy, and 36.7% were current smokers. Distributions of partners’ age, educational attainment, equivalent household income, and workplace SHS exposure differed among these four partners’ smoking categories. The prevalence of women’s quitting smoking after becoming aware of their pregnancy was higher among partners who quit smoking after becoming aware of the pregnancy compared with other partners.

**Table 1.** Characteristics of the partners of non-smoking pregnant women

	Total (*N* = 6,091)		Partners’ smoking status								*P*-value^a^

			Never smoked (*n* = 1,969)		Quit before pregnancy awareness (*n* = 1,690)		Quit after pregnancy awareness (*n* = 195)		Continued after pregnancy awareness (*n* = 2,237)		
	*n*	(%)	*n*	(%)	*n*	(%)	*n*	(%)	*n*	(%)	
Age											<0.001
≤29 years	1,491	(24.5)	533	(27.1)	293	(17.3)	61	(31.3)	604	(27.0)	
30–34 years	2,096	(34.4)	698	(35.5)	559	(33.1)	68	(34.9)	771	(34.5)	
35–39 years	1,537	(25.2)	454	(23.1)	495	(29.3)	44	(22.6)	544	(24.3)	
≥40 years	967	(15.9)	284	(14.4)	343	(20.3)	22	(11.3)	318	(14.2)	
Educational attainment											<0.001
University or higher	2,489	(40.9)	1,104	(56.1)	752	(44.5)	57	(29.2)	576	(25.8)	
College	1,289	(21.2)	367	(18.6)	371	(22.0)	55	(28.2)	496	(22.2)	
High school or lower	2,313	(38.0)	498	(25.3)	567	(33.6)	83	(42.6)	1,165	(52.1)	
Equivalent household income (/year)											<0.001
≥4.00 million Japanese yen	1,618	(26.6)	661	(33.6)	503	(29.8)	38	(19.5)	416	(18.6)	
3.00–3.99 million Japanese yen	1,287	(21.1)	438	(22.2)	356	(21.1)	52	(26.7)	441	(19.7)	
2.00–2.99 million Japanese yen	1,925	(31.6)	578	(29.4)	525	(31.1)	67	(34.4)	755	(33.8)	
≤1.99 million Japanese yen	1,261	(20.7)	292	(14.8)	306	(18.1)	38	(19.5)	625	(27.9)	
Secondhand smoke exposure at work											<0.001
Almost never or sometimes	3,170	(52.0)	1,451	(73.7)	1,047	(62.0)	83	(42.6)	589	(26.3)	
Almost every day	2,921	(48.0)	518	(26.3)	643	(38.0)	112	(57.4)	1,648	(73.7)	
Pregnant women’s smoking history											<0.001
Never smoked	3,911	(64.2)	1,563	(79.4)	1,117	(66.1)	94	(48.2)	1,137	(50.8)	
Quit before pregnancy awareness	1,478	(24.3)	342	(17.4)	507	(30.0)	33	(16.9)	596	(26.6)	
Quit after pregnancy awareness	702	(11.5)	64	(3.3)	66	(3.9)	68	(34.9)	504	(22.5)	

^a^Obtained using the chi-square test, comparing partners’ smoking status.

Table 2 presents the prevalence, ORs, and 95% CIs for continued smoking among partners who had been smokers when they became aware of the pregnancy. The prevalence of continued smoking was 92.0%. Age, education, and income were not associated with continued smoking among these partners. Workplace SHS exposure was associated with an increased risk of continued smoking: the multivariate-adjusted OR of workplace SHS exposure *almost every day* compared with *almost never or sometimes* was 2.08 (95% CI, 1.52–2.83). Pregnant women’s quitting smoking after—but not before—pregnancy awareness was associated with a decreased risk of partners’ continued smoking: the multivariate-adjusted ORs of women’s quitting smoking before pregnancy awareness and after pregnancy awareness compared with never smoking were 1.42 (95% CI, 0.94–2.15) and 0.57 (95% CI, 0.40–0.80), respectively.

**Table 2.** Prevalence, odds ratios (ORs) and 95% confidence intervals (CIs) for continued smoking among smoking partners when becoming aware of the pregnancy

	Continued smoking/ smoking partners	(%)	Age-adjusted OR (95% CI)		Multivariate-adjusted^a^ OR (95% CI)	
Total	2,237/2,432	(92.0)				
Age						
≤29 years	604/655	(90.8)	1.00		1.00	
30–34 years	771/839	(91.9)	1.15	(0.80–1.64)	1.18	(0.82–1.71)
35–39 years	544/588	(92.5)	1.25	(0.83–1.87)	1.29	(0.85–1.95)
≥40 years	318/340	(93.5)	1.46	(0.88–2.42)	1.55	(0.92–2.60)
Educational attainment						
University or higher	576/633	(91.0)	1.00		1.00	
College	496/551	(90.0)	0.91	(0.62–1.35)	0.92	(0.61–1.37)
High school or lower	1,165/1,248	(93.4)	1.41	(0.99–2.01)	1.25	(0.86–1.83)
Equivalent household income (/year)						
≥4.00 million Japanese yen	416/454	(91.6)	1.00		1.00	
3.00–3.99 million Japanese yen	441/493	(89.5)	0.80	(0.52–1.25)	0.80	(0.51–1.25)
2.00–2.99 million Japanese yen	755/822	(91.9)	1.09	(0.71–1.65)	1.03	(0.67–1.59)
≤1.99 million Japanese yen	625/663	(94.3)	1.59	(0.99–2.54)	1.47	(0.90–2.40)
Secondhand smoke exposure at work						
Almost never or sometimes	589/672	(87.7)	1.00		1.00	
Almost every day	1,648/1,760	(93.6)	2.14	(1.59–2.90)	2.08	(1.52–2.83)
Pregnant women’s smoking history						
Never smoked	1,137/1,231	(92.4)	1.00		1.00	
Quit before pregnancy awareness	596/629	(94.8)	1.46	(0.97–2.21)	1.42	(0.94–2.15)
Quit after pregnancy awareness	504/572	(88.1)	0.62	(0.44–0.86)	0.57	(0.40–0.80)

^a^Adjusted for all other variables in the table.

Table 3 presents the prevalence, ORs, and 95% CIs for indoor smoking among partners who continued to smoke after becoming aware of the pregnancy. The prevalence of indoor smoking was 30.2%. After multivariate adjustment, older age was associated with an increased risk of indoor smoking. Having a lower level of education was associated with an increased risk of indoor smoking: the multivariate-adjusted OR of having a high school education or lower compared with having a university education or higher was 1.51 (95% CI, 1.16–1.98). After multivariate adjustment, equivalent household income was not associated with indoor smoking. Workplace SHS exposure was associated with an increased risk of indoor smoking: the multivariate-adjusted OR of workplace SHS exposure *almost every day* compared with *almost never or sometimes* was 1.30 (95% CI, 1.02–1.66). Pregnant women’s quitting smoking after—but not before—pregnancy awareness was associated with an increased risk of partners’ indoor smoking: the multivariate-adjusted ORs of women’s quitting smoking before and after pregnancy awareness compared with never smoking were 0.97 (95% CI, 0.76–1.25) and 1.80 (95% CI, 1.39–2.32), respectively.

**Table 3.** Prevalence, odds ratios (ORs) and 95% confidence intervals (CIs) for indoor smoking among continued smoking partners after becoming aware of the pregnancy

	Indoor smoking/ continued smoking partners	(%)	Age-adjusted OR (95% CI)		Multivariate-adjusted^a^ OR (95% CI)	
Total	540/1,786	(30.2)				
Age						
≤29 years	131/434	(30.2)	1.00		1.00	
30–34 years	167/626	(26.7)	0.84	(0.64–1.10)	0.91	(0.69–1.20)
35–39 years	140/449	(31.2)	1.05	(0.79–1.40)	1.13	(0.84–1.52)
≥40 years	102/277	(36.8)	1.35	(0.98–1.85)	1.54	(1.10–2.14)
Educational attainment						
University or higher	111/493	(22.5)	1.00		1.00	
College	123/397	(31.0)	1.57	(1.16–2.12)	1.43	(1.05–1.95)
High school or lower	306/896	(34.2)	1.77	(1.38–2.28)	1.51	(1.16–1.98)
Equivalent household income (/year)						
≥4.00 million Japanese yen	94/360	(26.1)	1.00		1.00	
3.00–3.99 million Japanese yen	116/359	(32.3)	1.41	(1.02–1.95)	1.28	(0.92–1.79)
2.00–2.99 million Japanese yen	179/610	(29.3)	1.24	(0.92–1.67)	1.09	(0.80–1.48)
≤1.99 million Japanese yen	151/457	(33.0)	1.47	(1.08–2.01)	1.22	(0.88–1.69)
Secondhand smoke exposure at work						
Almost never or sometimes	121/472	(25.6)	1.00		1.00	
Almost every day	419/1,314	(31.9)	1.40	(1.10–1.78)	1.30	(1.02–1.66)
Pregnant women’s smoking history						
Never smoked	248/922	(26.9)	1.00		1.00	
Quit before pregnancy awareness	131/481	(27.2)	1.00	(0.78–1.28)	0.97	(0.76–1.25)
Quit after pregnancy awareness	161/383	(42.0)	1.98	(1.54–2.54)	1.80	(1.39–2.32)

^a^Adjusted for all other variables in the table.

## DISCUSSION

The present study examined social factors associated with continued smoking and indoor smoking among the partners of non-smoking pregnant women in Japan. After becoming aware of the pregnancy, 92% of smoking partners continued to smoke. Workplace SHS exposure was associated with an increased risk of continued smoking, and pregnant women’s quitting smoking after—but not before—pregnancy awareness was associated with a decreased risk of continued smoking among partners. About one-third of the partners who continued to smoke after becoming aware of the pregnancy smoked indoors. Having a lower level of education, being exposed to SHS in the workplace, and pregnant women’s quitting smoking after pregnancy awareness were associated with increased risks of indoor smoking among partners who continued to smoke.

Among partners who had smoked when they became aware of the pregnancy, 92% continued smoking after pregnancy awareness. One previous study in Japan reported that the corresponding prevalence was 90.4%.^16^ Pregnancy has been considered a “teachable moment” and an especially motivating time for women because of their sense of the fetal effects of smoking and the social pressure not to smoke during pregnancy.^17^ These women’s partners, however, often experience less social pressure and immediate motivation to quit smoking compared with pregnant women.^18^ Enhancing partners’ motivation to quit is necessary to reduce their continued smoking during pregnancy.

Workplace SHS exposure was associated with increased risks of continued smoking and indoor smoking. SHS exposure has been one of the most frequently observed factors associated with continued smoking among pregnant women.^6^ The present study has also demonstrated this association among the partners of non-smoking pregnant women. Many adults spend most of their day in a workplace environment, and the workplace can reinforce social support networks and peer influences among coworkers.^19^ Considerable evidence indicates that smoke-free policies reduce tobacco use when implemented in the workplace.^20^^,^^21^ One study in Japan has also suggested that, among smoking fathers living with their children aged ≤6 years, the awareness of the need to prevent SHS exposure at home may increase when they feel that smoking is not accepted in their workplace.^22^ Considering men’s work orientation in the Japanese cultural context, the partners of pregnant women who are exposed to SHS at work may tend to have a greater acceptance of smoking, which leads to higher rates of continued smoking and indoor smoking. At the time of the study (2013–2017), there was no national legislation prohibiting indoor smoking in Japan.^23^ However, the revised Health Promotion Act, which prohibits smoking in public facilities, was subsequently implemented in stages and came into full force in April 2020. This workplace improvement can be expected to decrease workplace SHS exposure, which may help the partners of pregnant women to quit or refrain from smoking.

Pregnant women’s smoking cessation after pregnancy awareness was associated with a decreased risk of partners’ continued smoking, whereas women’s smoking cessation before pregnancy awareness was not associated with partners’ continued smoking. These results suggest that the timing of women’s smoking cessation affects the association of this change in health behavior with partners’ continued smoking. It is well known that health behaviors are often concordant across couples.^24^^,^^25^ This concordance results partly from assortative mating (couples with similar characteristics are more likely to marry), but it may also reflect spousal influence on each other’s health behaviors.^26^ People are more likely to make a positive health behavior change if their partner does so as well, with a positive shift in partner’s health behavior having a stronger effect than the partner having consistently healthy behavior in that domain.^27^ Spouse’s smoking cessation has been reported to decrease an individual’s chances of smoking by 67%.^28^ Couples may decide to change together, or successful behavior change in one partner may encourage the other to try to change their behavior. The partners of women who never smoked or quit smoking before becoming aware of their pregnancy may have fewer motivating opportunities to quit. One intervention program directed specifically at the partners of pregnant women has demonstrated success with partner smoking cessation.^29^ Taken together, our findings indicate the need for the development of interventions providing the partners of pregnant women with information and education on the importance of their smoking cessation during pregnancy. On the other hand, among partners who continued to smoke during the pregnancy, women’s smoking cessation after pregnancy awareness was associated with an increased risk of indoor smoking. A possible explanation for this association is that smoking partners tended to refrain from indoor smoking before women’s pregnancy if the women had never smoked or quit smoking before becoming aware of their pregnancy, whereas they did not do so if the women quit smoking only after becoming aware of their pregnancy. Although we could not examine this explanation because we do not have data on indoor or outdoor smoking before pregnancy among partners who continued to smoke after becoming aware of the pregnancy, partners who continue to smoke after women successfully quit should receive attention to protect pregnant women from SHS exposure at home.

Lower levels of education were associated with indoor smoking among the partners of non-smoking pregnant women. This finding is consistent with previous reports that fathers’ lower education is associated with children’s exposure to SHS at home.^30^ Education conveys factual health-related knowledge and increases cognitive skills, both of which affect health-promoting decisions.^31^^,^^32^ Enhancing knowledge about the health risks of SHS exposure during pregnancy, especially for less educated partners of pregnant women, would likely be helpful in reducing the association between education and indoor smoking. World Health Organization guidelines recommend that health care providers should provide pregnant women and their partners with advice and information about the health risks of SHS exposure as well as strategies to reduce SHS in the home.^33^

The present findings have implications for the design of effective interventions aimed at reducing SHS exposure among pregnant women. Most smoking partners of pregnant women continued to smoke even after becoming aware of the pregnancy, and about one-third of them did so inside the pregnant women’s residence, which can have harmful effects on pregnant women and their fetuses. Pregnancy is an opportunity to directly engage not only women, but also their partners, and to explain the need to protect the unborn child from the harms of SHS.^33^ Pregnancy is also an opportunity to encourage both women and their partners to quit smoking while they are still relatively young and healthy and to explain that quitting earlier in life is associated with greater health benefits.^33^ Little attention has been paid to addressing smoking among the partners of pregnant women, although these partners play a central role in creating a smoke-free environment for pregnant women. Furthermore, there are few effective smoking cessation interventions that include partners or examine partners’ smoking during pregnancy.^18^^,^^34^^,^^35^ Considering the social factors observed in the present study may be helpful in developing effective partner-focused interventions.

The present study has several limitations. First, the study was conducted in one of the 47 prefectures in Japan; therefore, generalizability is limited. The prevalence of smoking in Miyagi Prefecture is similar to that reported in a national survey in 2016: 33.4% among men and 9.7% among women in Miyagi Prefecture compared with 31.1% among men and 9.5% among women in the national survey.^36^ National surveys have reported that the percentage of men who were exposed to workplace SHS almost every day was about 20.0% from 2013 to 2017,^37^ which is lower than the finding of 48.0% in the present study. Second, partners’ smoking status was assessed using pregnant women’s self-report data, introducing the risk of reporting bias associated with lack of knowledge. Additionally, for about one-fifth of the partners who continued to smoke after becoming aware of the pregnancy, there was no information on whether they smoked indoors or outdoors. Third, it is possible that some women underreported their own active smoking because active smoking has become more socially unacceptable.^38^^,^^39^ Fourth, smoking cessation in middle/late pregnancy was not considered because we examined the smoking status of pregnant women and their partners in early pregnancy. However, many women who smoked before pregnancy quit smoking on their own shortly after becoming pregnant.^40^ Finally, the questionnaire did not state that heated tobacco product use should be included in smoking. However, a study in Japan reported that the prevalence of heated tobacco use was 0.2% in 2015, 0.8% in 2016, and 3.7% in 2017,^41^ which suggests that heated tobacco product use likely had only a small effect in the present study.

In conclusion, most smoking partners of non-smoking pregnant women continued to smoke even after becoming aware of the pregnancy, and about one-third of them smoked indoors. Workplace SHS exposure was associated with an increased risk of continued smoking, and pregnant women’s quitting smoking after—but not before—pregnancy awareness was associated with a decreased risk of continued smoking. Among those who continued to smoke, lower education, workplace SHS exposure, and pregnant women’s quitting smoking after pregnancy awareness were associated with increased risks of indoor smoking. These factors should be considered when designing interventions to prevent partners’ continued smoking and SHS exposure among pregnant women, which is important for maternal and child health as well as for promoting the health of the partners themselves.

## APPENDIX A. SUPPLEMENTARY DATA

eTable 1. Details of questionnaires used in the analysis
