# Supplementary material for: Social Predictors of Continued and Indoor Smoking Among Partners of Non-smoking Pregnant Women: The TMM BirThree Cohort Study
Source: J Epidemiol. 2021 Dec 5;31(12):635–41. doi: 10.2188/jea.JE20200313 (PMC8593571; doi:10.2188/jea.JE20200313)
Supplement: Supplementary file 1 [file je-31-635-s001.pdf]

eTable 1. Details of questionnaires used in the analysis

	Questionnaire 1	Questionnaire 2	Questionnaire 3
Respondents	Partners	Pregnant women	Pregnant women
Timing	Enrolment (early pregnancy[a])	Enrolment (early pregnancy[a])	Middle pregnancy[b]
Variables	Age<br>Educational attainment<br>Secondhand smoke exposure at work	Household members<br>Pregnant women's smoking status<br>Partners' smoking status<br>Number of household smokers<br>Number of smokers inside the residence	Household income

[a]Early pregnancy was defined as <14 weeks of gestation.
[b]Middle pregnancy was defined as 14–27 weeks of gestation.
